# Virulence-Associated Genomic Architecture of Canine Otitis Externa-Derived *Pseudomonas aeruginosa* Isolates from Hungary

**DOI:** 10.3390/vetsci13070664

**Published:** 2026-07-08

**Authors:** Mercédesz Adrienn Veres, Zsófia Anna Tóth, Enikő Illés, Patrik Mag, Eszter Kaszab, Enikő Fehér, Ákos Jerzsele, Ádám Kerek

**Affiliations:** 1 Department of Pharmacology and Toxicology, University of Veterinary Medicine Budapest, István Utca 2, H-1078 Budapest, Hungary; veres.a.mercedesz@univet.hu (M.A.V.); toth.zsofia.anna@student.univet.hu (Z.A.T.); illes.eniko@student.univet.hu (E.I.); mag.patrik@univet.hu (P.M.); jerzsele.akos@univet.hu (Á.J.); 2 National Laboratory of Infectious Animal Diseases, Antimicrobial Resistance, Veterinary Public Health and Food Chain Safety, University of Veterinary Medicine Budapest, István Utca 2, H-1078 Budapest, Hungary; kaszab.eszter@univet.hu (E.K.); feher.eniko@univet.hu (E.F.); 3 Department of Bioinformatics, One Health Institute, Faculty of Health Sciences, University of Debrecen, Nagyerdei Krt. 98, H-4032 Debrecen, Hungary; 4 Department of Microbiology and Infectious Diseases, University of Veterinary Medicine Budapest, István Utca 2, H-1078 Budapest, Hungary; 5 National Laboratory of Virology, Szentágothai Research Centre, University of Pécs, Ifjúság Útja 20, H-7624 Pécs, Hungary

**Keywords:** canine otitis externa, *Pseudomonas aeruginosa*, virulome, whole-genome sequencing, type III secretion system, biofilm, multilocus sequence typing, One Health

## Abstract

**Background:** Canine otitis externa is a clinically relevant niche where *Pseudomonas aeruginosa* may persist under inflammatory, antimicrobial, and biofilm-favoring conditions. This study characterized the virulome and population structure of canine otitis externa-derived *P. aeruginosa* isolates from Hungary. **Methods:** Of 110 isolates previously subjected to minimum inhibitory concentration testing, 70 phenotypically selected isolates underwent long-read whole-genome sequencing and were included in virulence-factor analysis. Virulence-associated genes were detected using the Virulence Factor Database with an 80% identity and 80% coverage threshold and were interpreted by functional systems, including motility, adhesion, biofilm formation, secretion systems, siderophores, quorum sensing, secreted enzymes, and type III secretion system effectors. **Results:** The isolates carried a broad and highly conserved virulence-associated backbone. Genes related to alginate production, type IV pili, flagellar motility, pyochelin, phenazine biosynthesis, Xcp secretion, type VI secretion, and proteolytic enzymes were widely distributed. Type III secretion effector genes were variable: *exoT* was detected in 68/70 isolates, *exoY* in 63/70, *exoS* in 50/70, and *exoU* in 15/70. MLST indicated a genetically diverse population, and virulome size showed no strong correlation with the modified multiple antimicrobial resistance index. **Conclusions:** These findings support integrated genomic surveillance of virulence and antimicrobial resistance in canine otitis-associated *P. aeruginosa*.

## Simple Summary

Ear infections are common in dogs and can be difficult to treat when they involve *Pseudomonas aeruginosa*, a bacterium that can survive in inflamed tissues, form protective communities called biofilms, and tolerate antimicrobial treatment. In this study, we examined the genetic background of *P. aeruginosa* bacteria isolated from dogs with outer ear infections in Hungary. Our aim was to identify genes that may help these bacteria attach to tissues, move, form biofilms, acquire nutrients, damage host tissues, and persist during infection. We analyzed 70 bacterial genomes and found that most isolates carried a broad set of genes linked to survival and disease-causing potential. Several important genes were highly conserved, while others, especially genes related to toxin delivery, varied between isolates. The bacteria also represented a genetically diverse population, suggesting that difficult canine ear infections may be caused by multiple bacterial lineages rather than a single dominant type. These findings show that treatment difficulty is not only related to antimicrobial resistance, but also to bacterial traits that support persistence and tissue damage. Understanding these features may help improve veterinary diagnosis, infection monitoring, and future treatment strategies for companion animals.

## 1. Introduction

Otitis externa is among the most frequent inflammatory disorders encountered in companion-animal practice and is clinically important not only because it causes pain, pruritus and reduced quality of life, but also because chronic and recurrent cases may progress to structural ear-canal changes, otitis media and, in severe cases, surgical intervention. The disease is rarely a simple primary bacterial process. Instead, it is usually shaped by primary, predisposing and perpetuating factors, including allergy, anatomical conformation, moisture, canal stenosis and secondary microbial overgrowth [1,2,3,4,5].

*Pseudomonas aeruginosa* is particularly relevant in chronic or treatment-refractory canine otitis externa. It is an opportunistic, environmentally resilient Gram-negative bacterium that can persist in moist niches, form biofilm on biotic and abiotic surfaces, and tolerate many antimicrobial or antiseptic pressures. These features make it difficult to eradicate from inflamed ear canals and veterinary clinical environments [1,6,7,8,9,10].

The clinical challenge posed by *P. aeruginosa* is often framed through antimicrobial resistance, and appropriately so: the species combines low outer-membrane permeability, inducible beta-lactamase activity, efflux systems, porin-related changes and acquired determinants that may restrict systemic and topical treatment options [11,12,13,14,15]. However, therapeutic failure in canine otitis is not explained by resistance alone. Persistent infection is also influenced by adhesion, motility, secretion systems, toxin delivery, extracellular enzymes, iron acquisition, quorum sensing and biofilm matrix production. These virulence-associated systems may promote colonization of a damaged ear canal, impair host clearance, exacerbate tissue injury and protect bacterial communities from treatment [1,9,15,16,17].

Several virulence systems are central to the biology of *P. aeruginosa*. Type IV pili and flagella contribute to adhesion, twitching motility and early surface colonization. Alginate and related mucoid biofilm pathways support persistence in protected bacterial communities. Siderophore systems, including pyoverdine and pyochelin, facilitate iron acquisition in host-restricted environments. The Las and Rhl quorum-sensing networks coordinate extracellular enzyme, phenazine and rhamnolipid production. The type III secretion system (T3SS) is especially relevant because its effector repertoire, including ExoS, ExoU, ExoT and ExoY, can influence cytotoxicity, epithelial injury and inflammatory outcomes [9,15,16].

Despite increasing availability of veterinary whole-genome sequencing data, virulence-focused analyses of canine otitis-associated *P. aeruginosa* remain limited compared with resistance-focused surveillance. Existing companion-animal studies indicate that ear-canal isolates may include genetically diverse lineages and clinically relevant resistance profiles, but the extent to which these isolates share a conserved virulence backbone or differ in accessory virulence determinants remains incompletely defined [7,18,19,20].

The objective of this study was therefore to characterize the virulence-associated genomic architecture of a phenotypically selected whole-genome sequenced subset of canine otitis externa-derived *P. aeruginosa* isolates from Hungary. Specifically, we aimed to determine the distribution of VFDB-detected virulence systems, identify variable and clinically relevant effector patterns, describe MLST-based population structure, and explore whether virulome burden was associated with phenotypic antimicrobial resistance burden measured by a modified multiple antimicrobial resistance (MAR) index.

## 2. Materials and Methods

### 2.1. Study Design and Isolate Collection

This retrospective laboratory study used canine otitis externa-associated *P. aeruginosa* isolates recovered from routine veterinary diagnostic submissions in Hungary. The broader phenotypic dataset comprised 110 isolates subjected to broth microdilution minimum inhibitory concentration (MIC) testing against 14 antimicrobial or antiseptic agents. For genomic analysis, 70 isolates were selected after completion of MIC testing to maximize phenotypic heterogeneity within the collection. Selection was based on the modified MAR index and the distribution of MIC-derived susceptibility patterns across clinically relevant antimicrobial groups, including fluoroquinolones, aminoglycosides, beta-lactams and other agents included in the phenotypic panel. The aim was to include isolates spanning the observed resistance-burden range and divergent MIC profiles rather than to create a random or prevalence-representative sample. No formal stratified random sampling with fixed numerical quotas was applied. Because genomic data were not available before sequencing, redundant clones were not excluded during selection; potential clonal relatedness was instead evaluated post hoc using MLST and core-genome phylogenetic analysis. All 70 sequenced isolates were included in the present virulome analysis.

### 2.2. Phenotypic Background and Resistance-Burden Metric

The full phenotypic dataset and the selected WGS subset were derived from the same canine otitis externa-associated *Pseudomonas aeruginosa* isolate collection as the companion MIC-focused study. MIC testing was performed by broth microdilution. MIC interpretation followed Clinical and Laboratory Standards Institute (CLSI) and European Committee on Antimicrobial Susceptibility Testing (EUCAST) criteria where available, supplemented by literature-based thresholds when official veterinary breakpoints were not available [21,22,23,24]. For this virulence-focused manuscript, MIC values were not reinterpreted as the primary outcome. Instead, they were used to calculate a modified MAR index for isolates in the WGS subset. The index was calculated as the number of antimicrobial agents exceeding predefined resistance or non-susceptibility thresholds divided by the number of agents with interpretable thresholds. This metric was used only as a descriptive resistance-burden covariate.

### 2.3. DNA Extraction, Sequencing and Assembly

Bacterial DNA was extracted from revived *P. aeruginosa* isolates using the Quick-DNA Fungal/Bacterial Miniprep Kit (Zymo Research Corp., Irvine, CA, USA), following the manufacturer instructions. DNA concentration was measured using a Qubit 4.0 Fluorometer (Thermo Fisher Scientific, Waltham, MA, USA). Sequencing libraries were prepared using the Native Barcoding Kit 24 V14 (Oxford Nanopore Technologies Ltd., Oxford, UK) and loaded onto R10.4.1 MinION flow cells. Sequencing was performed on a MinION Mk1B instrument (Oxford Nanopore Technologies Ltd., Oxford, UK). Basecalling and demultiplexing were performed in super-accuracy mode using Dorado integrated into MinKNOW.

Raw long reads were filtered and trimmed with NanoLyse v1.2.1 and NanoFilt v2.8.0. Reads with mean quality below 8 or length below 500 bp were removed, and 50 bp was trimmed from both read ends to reduce residual adapter sequences. Read quality was summarized with NanoPlot v1.43.0. De novo assembly was performed with Flye v2.9.5, followed by polishing with minimap2 v2.28, racon v1.5.0 and medaka v2.0.1. Assembly quality was evaluated using QUAST v5.0.2, BUSCO v5.8.2 and CheckM v1.2.2. Multilocus sequence typing profiles were generated using the mlst pipeline [25,26,27,28,29,30,31]. Hybrid short-read validation was not performed. Although hybrid assemblies combining long- and short-read sequencing can be advantageous in some applications, the long-read data generated in this study produced highly contiguous, high-quality assemblies that adequately addressed the genome-wide screening objectives of the work. Therefore, additional short-read sequencing was not considered necessary for the primary virulome analysis. Nevertheless, gene absence calls, particularly for variable loci, were interpreted cautiously.

### 2.4. Virulence-Factor Detection and Functional Annotation

Virulence-associated genes were identified from assembled genomes using ABRicate v1.0.1 against the Virulence Factor Database (VFDB; database accessed on 5 May 2026). Hits were retained when sequence identity and coverage were both at least 80%. These thresholds were selected as conservative screening criteria to reduce the inclusion of short, low-coverage or weakly similar matches while retaining homologous virulence-associated loci that may show sequence variation among clinical isolates. Similar identity- and coverage-based filtering approaches are commonly applied in bacterial WGS pipelines using ABRicate or related BLAST-based screening tools for resistance and virulence gene detection [32]. Detected loci were grouped into functional systems based on VFDB product annotation, including alginate and mucoid biofilm matrix, alginate regulation, type IV pili, flagellar motility, T3SS, T3SS effectors, type VI secretion system, Xcp type II secretion system, pyochelin and pyoverdine siderophore systems, phenazine/pyocyanin pathways, rhamnolipid biosurfactant systems, quorum-sensing genes, LPS/surface-antigen systems, secreted proteases and phospholipase C. Because VFDB reports individual loci from multi-gene systems, interpretation emphasized system-level presence rather than overinterpreting single-gene carriage as a direct measure of phenotype.

No additional single-gene PCR confirmation was performed, because the objective of the study was genome-wide virulome characterization based on long-read WGS data rather than diagnostic validation of selected loci. Targeted PCR, RT-qPCR or functional assays should be considered in future studies to validate selected genes and determine whether detected loci are expressed under otitis-relevant conditions.

### 2.5. Core-Genome Phylogenetic Analysis

All assemblies were annotated using Prokka v1.14.6 with lineage-specific settings (--genus Pseudomonas --usegenus). The core genome was identified with Panaroo v1.6.0 using strict cleaning mode (--clean-mode strict). Phylogenetic relationships among isolates were reconstructed with IQ-TREE v2.4.0 using the core-genome alignment as input and automatic model selection (-m MFP). The best-fit model selected according to the Bayesian information criterion was GTR+F+I+R10. The resulting phylogenetic tree was visualized with FigTree v1.4.4 and further edited in Inkscape v1.4.2 to improve readability.

### 2.6. Statistical Analysis

Analyses were performed in Python v. 3.11 using pandas, NumPy, SciPy, matplotlib and seaborn. Descriptive statistics were calculated for gene and category prevalence. The number of distinct virulence-associated genes and the number of functional virulence categories per isolate were used as descriptive virulome-burden metrics. MLST diversity was summarized by sequence-type counts. T3SS effector profiles were summarized using *exoS*, *exoU*, *exoT* and *exoY* carriage. Spearman correlation was used to test the association between total virulence-gene count and modified MAR index. The Mann–Whitney U test was used to compare modified MAR indices between *exoU*-positive and *exoU*-negative isolates. Statistical tests were considered exploratory, and *p* values were interpreted cautiously because the WGS subset was selected rather than randomly sampled.

## 3. Results

### 3.1. Composition and Quality of the Virulome Dataset

All 70 isolates selected for WGS were included in the final virulome analysis. Therefore, all denominator-based virulence prevalence calculations were performed using 70 isolates.

**Figure 1:**
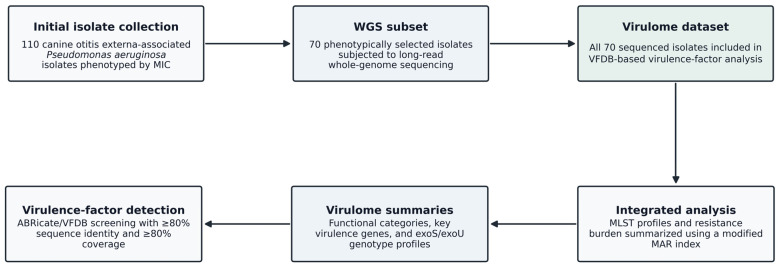
Study design and virulome-analysis workflow. The phenotypic isolate collection included 110 canine otitis externa-associated *Pseudomonas aeruginosa* isolates subjected to minimum inhibitory concentration testing. A phenotypically selected subset of 70 isolates underwent long-read whole-genome sequencing, and all 70 sequenced isolates were included in VFDB-based virulence-factor analysis. MIC, minimum inhibitory concentration; WGS, whole-genome sequencing; VFDB, Virulence Factor Database; MLST, multilocus sequence typing; MAR, multiple antimicrobial resistance.

The sequenced assemblies had a median genome size of 6.57 Mbp and median GC content of 66.27%. Median CheckM completeness was 99.68% and median contamination was 0.14%. These values support the use of the currently analyzed genomes for broad virulome screening (Table 1). The overall study design and filtering workflow from the phenotypic isolate collection to the final virulome dataset are summarized in Figure 1.

### 3.2. A Conserved Virulence Backbone Was Present Across the Sequenced Isolates

**Figure 2:**
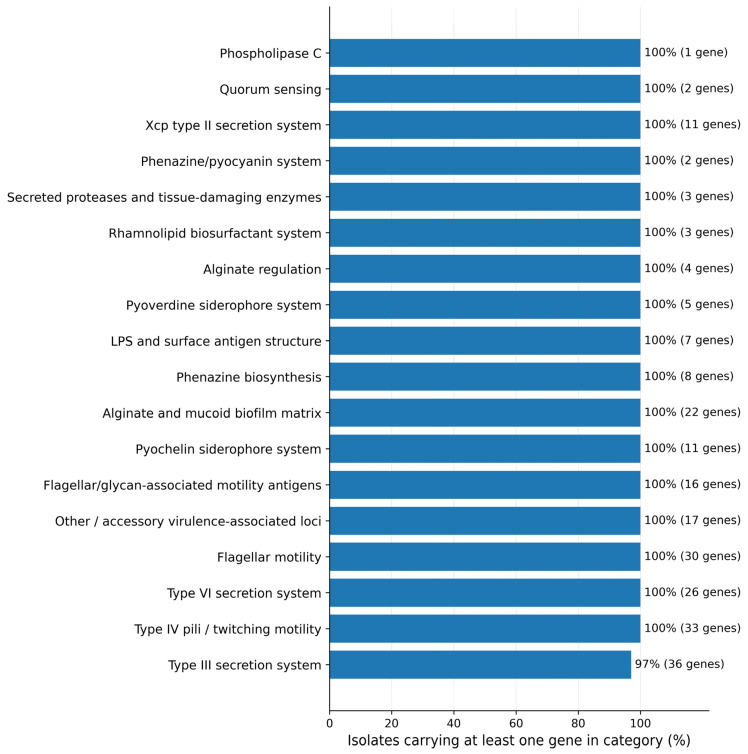
Virulence-associated functional systems in the whole-genome sequenced subset. Bars indicate the percentage of canine otitis externa-derived *Pseudomonas aeruginosa* isolates carrying at least one VFDB-detected gene in each functional category. Values in parentheses denote the number of distinct genes assigned to each category. Most systems were detected in all isolates, whereas the type III secretion system showed slightly lower prevalence. VFDB, Virulence Factor Database.

The VFDB screen identified a broad virulence-associated repertoire. The median number of distinct VFDB-detected genes per isolate was 230 (interquartile range 226–232). Most major systems were near-universal, including alginate-associated loci, type IV pili, flagellar motility genes, type VI secretion components, pyochelin biosynthesis genes, Xcp secretion genes, phenazine biosynthesis genes and secreted protease determinants (Table 2; Figure 2). This pattern indicates that canine otitis-associated isolates carry a conserved pathogenicity framework rather than isolated virulence features.

### 3.3. Biofilm, Adhesion and Motility Systems Were Broadly Distributed

**Figure 3:**
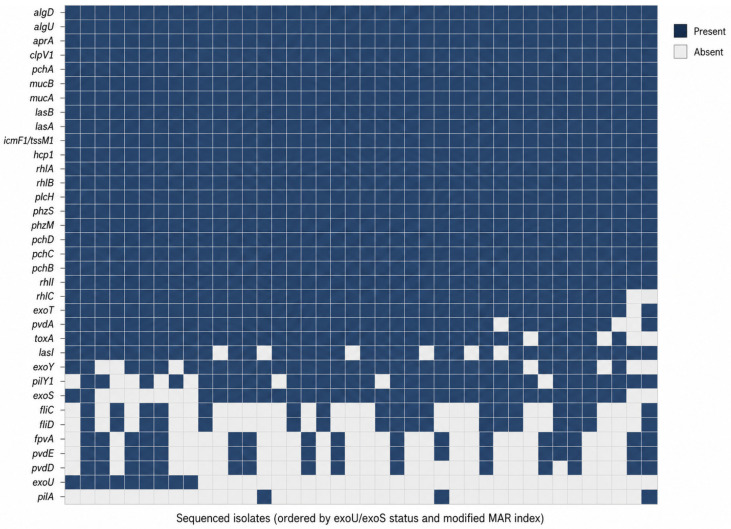
Distribution of clinically relevant and variable virulence-associated genes in the whole-genome sequenced subset. Heatmap showing the presence and absence of selected clinically relevant and variable virulence-associated genes across canine otitis externa-derived *Pseudomonas aeruginosa* isolates. Each column represents one sequenced isolate and each row represents one gene. Dark-colored cells indicate gene presence, whereas light-colored cells indicate gene absence. Isolates are ordered according to *exoU*/*exoS* status and modified multiple antimicrobial resistance (MAR) index to facilitate comparison of major type III secretion system effector profiles and phenotypic resistance burden. The heatmap illustrates a broadly conserved virulence-associated genomic backbone together with variability in selected effector, siderophore-associated, motility-associated, and pili-associated loci. MAR, multiple antimicrobial resistance.

Genes linked to alginate biosynthesis and regulation were highly prevalent. This included alginate structural and regulatory loci such as *algA*, *algC*, *algD*, *algG*, *algU*, *algR* and *muc*-associated genes (Table 3; Figure 3). These findings are relevant because chronic otitis externa provides a biofilm-favoring environment where alginate and extracellular matrix production can increase persistence and reduce antimicrobial exposure. Adhesion and motility systems were also widespread: type IV pilus-associated genes and flagellar genes occurred in nearly all analyzed isolates, consistent with a genomic background capable of twitching motility, surface exploration, epithelial interaction and early biofilm establishment (Table 2; Figure 2).

### 3.4. T3SS Effector Profiles Showed Clinically Relevant Heterogeneity

**Figure 4:**
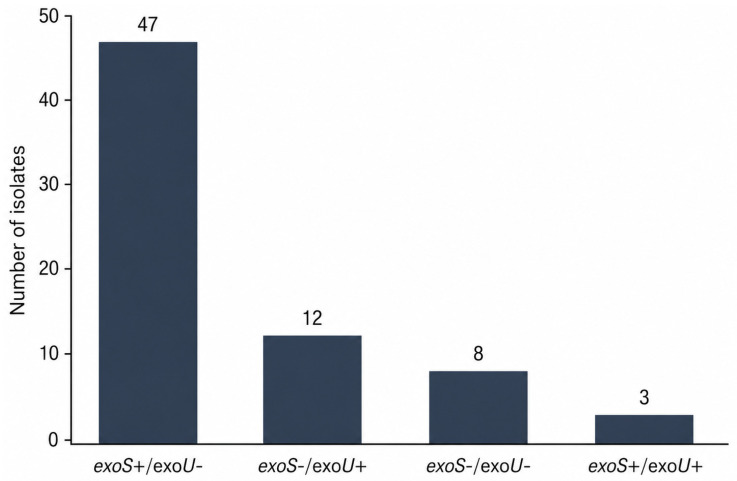
T3SS effector genotype profiles in the whole-genome sequenced subset. Bar chart showing the distribution of major type III secretion system (T3SS) effector genotype profiles among canine otitis externa-derived *Pseudomonas aeruginosa* isolates. Bars represent the number of isolates carrying each *exoS*/*exoU* genotype combination, and values above the bars indicate isolate counts. The *exoS*+/*exoU*− profile was the most frequent, whereas *exoS*−/*exoU*+, *exoS*−/*exoU*−, and *exoS*+/*exoU*+ profiles were less common. This distribution indicates that *exoS*-positive/*exoU*-negative isolates predominated in the dataset, while *exoU*-positive genotypes were present in a smaller subset. T3SS, type III secretion system.

The type III secretion system core machinery was broadly conserved, but effector carriage differed among isolates. *exoT* was detected in 68/70 isolates, *exoY* in 63/70, *exoS* in 50/70 and *exoU* in 15/70 (Table 3; Figure 4). The dominant profile was *exoS*-positive/*exoU*-negative, while an *exoU*-positive subset was also present (Figure 4). Because ExoU is frequently interpreted as a marker of a more cytotoxic *P. aeruginosa* phenotype, its detection in a subset of canine otitis isolates is a potentially important finding. However, the presence of *exoU* should not be interpreted as direct evidence of active toxin expression, secretion or cytotoxic phenotype. Functional cytotoxicity assays, epithelial-cell infection models or transcriptomic analysis under ear-canal-like conditions would be required to confirm the biological consequences of these effector profiles.

### 3.5. Iron Acquisition, Extracellular Enzymes and Quorum-Sensing-Associated Genes Were Common

Siderophore-associated genes were frequent, especially pyochelin-system genes. Pyoverdine-associated loci were also detected, although several pyoverdine biosynthesis/receptor genes showed more variable distribution (Table 2 and Table 3; Figure 3). Secreted tissue-damaging enzymes, including *lasA*, *lasB* and *aprA*, were widely detected. Quorum-sensing-associated loci, including *rhlI*/*rhlR* and *lasI*/*lasR*-related hits, were also present in much of the dataset (Table 3). Together, these systems support the biological plausibility that canine otitis isolates can coordinate extracellular enzyme production, iron acquisition, biofilm development and competitive persistence within the inflamed ear canal.

### 3.6. MLST Indicated a Diverse Population Rather than a Single Clonal Expansion

MLST analysis showed multiple sequence types among the whole-genome sequenced isolates (Table 4). A substantial fraction of isolates had novel or incomplete ST calls, which may reflect allelic novelty, assembly fragmentation affecting one or more loci, or database limitations. The diversity of STs argues against the dataset being driven by a single clonal expansion and supports interpretation as a heterogeneous clinical collection. The high number of novel or incomplete MLST profiles should be interpreted conservatively. Given the high overall assembly completeness and low contamination values, this pattern is unlikely to reflect a generalized assembly-quality problem across the dataset. However, incomplete recovery, local assembly uncertainty or sequence divergence affecting one or more MLST loci cannot be excluded for individual isolates. In addition, the available *P. aeruginosa* MLST database may not fully capture the allelic diversity of veterinary otitis-associated lineages. Therefore, MLST was interpreted as a descriptive typing layer rather than as the sole basis for population-structure inference, and isolate relatedness was further evaluated using core-genome phylogenetic analysis.

### 3.7. Core-Genome Phylogeny Confirmed a Genetically Heterogeneous Isolate Collection

**Figure 5:**
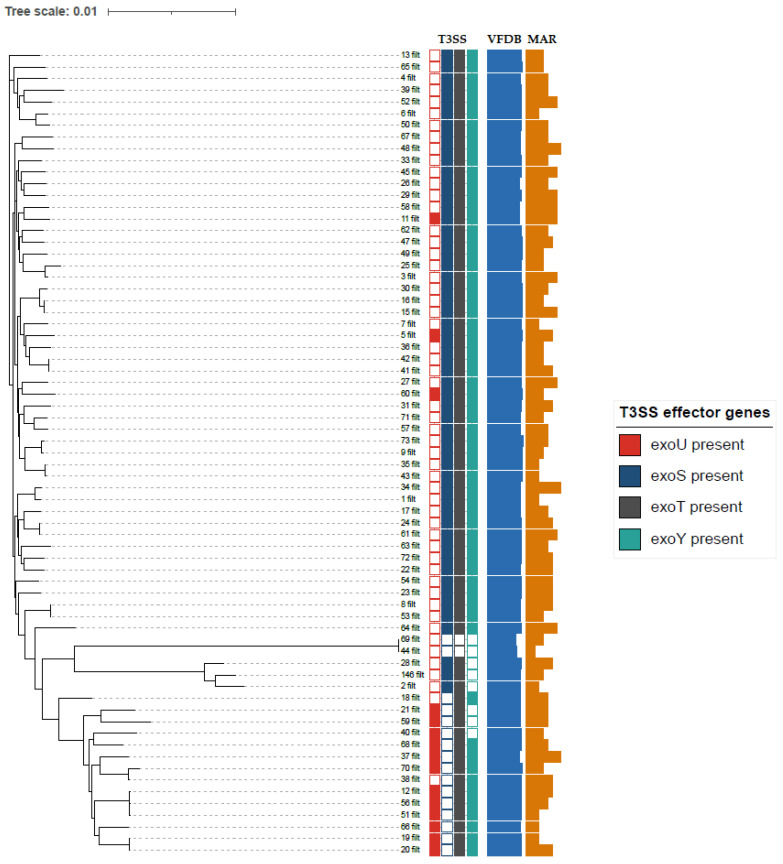
Core-genome phylogenetic relationships among canine otitis externa-derived *Pseudomonas aeruginosa* isolates. The tree was reconstructed from the core-genome alignment using IQ-TREE with automatic model selection. The best-fit model selected according to the Bayesian information criterion was GTR+F+I+R10. The adjacent annotation tracks indicate the presence of type III secretion system effector genes (*exoU*, *exoS*, *exoT* and *exoY*). Blue bars indicate VFDB-detected virulence-gene count, and orange bars indicate the modified multiple antimicrobial resistance (MAR) index. The phylogeny shows that the isolates were distributed across multiple branches rather than forming a single outbreak-like clonal cluster.

Core-genome phylogenetic analysis provided a higher-resolution overview of isolate relatedness than MLST alone (Figure 5). The tree showed that the sequenced isolates did not form a single outbreak-like cluster, but were distributed across multiple phylogenetic branches, supporting the interpretation of a genetically heterogeneous clinical collection. Isolates with novel or incomplete MLST profiles were not confined to one branch, suggesting that these calls did not reflect a single technical artifact or one narrowly defined lineage. The distribution of *exoU*-positive isolates across the tree further indicated that T3SS effector carriage was not limited to a single dominant clonal background. Together, these findings support the use of core-genome phylogeny as the primary population-structure framework, with MLST retained as a complementary descriptive typing layer.

### 3.8. Virulome Burden Was Not Strongly Coupled to Phenotypic Resistance Burden

**Figure 6:**
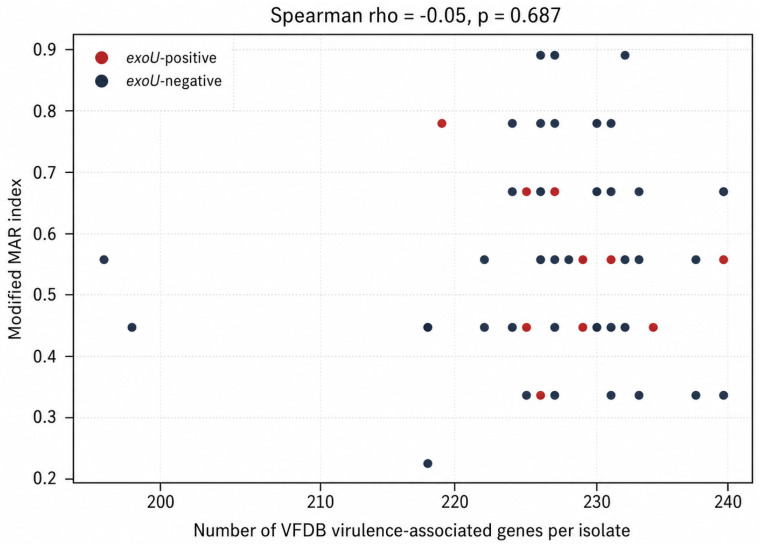
Relationship between virulome size and phenotypic resistance burden in the whole-genome sequenced subset. Scatter plot showing the relationship between the total number of VFDB-detected virulence-associated genes per isolate and the modified multiple antimicrobial resistance (MAR) index in canine otitis externa-derived *Pseudomonas aeruginosa* isolates. Each point represents one isolate. Red points indicate *exoU*-positive isolates and blue points indicate *exoU*-negative isolates. The weak and non-significant Spearman correlation (ρ = −0.05, *p* = 0.687) indicates that virulome size was not strongly associated with phenotypic resistance burden in this dataset. MAR, multiple antimicrobial resistance; VFDB, Virulence Factor Database.

To explore whether broader virulence-gene carriage tracked with phenotypic resistance burden, total VFDB gene count was compared with the modified MAR index in the WGS subset. This analysis integrated isolate-level virulome burden, phenotypic resistance burden and *exoU* status to evaluate whether broader virulence-gene carriage coincided with resistance-associated phenotypic profiles. The association was weak (Spearman ρ = −0.05, *p* = 0.687; Table 5; Figure 6). *exoU*-positive isolates had a median modified MAR index of 0.56, compared with 0.56 among *exoU*-negative isolates (Mann–Whitney U test, *p* = 0.549; Table 5). These exploratory results suggest that resistance burden and virulence-gene carriage should be interpreted as complementary rather than interchangeable features.

## 4. Discussion

This study provides a virulence-focused genomic analysis of canine otitis externa-associated *P. aeruginosa* isolates from Hungary and extends the companion phenotypic antimicrobial susceptibility dataset by adding a second, clinically relevant dimension of pathogenic potential. The main finding was that the analyzed isolates carried a broad and largely conserved virulence-associated genomic backbone, including genes involved in alginate and biofilm matrix formation, type IV pili, flagellar motility, secretion systems, siderophore-mediated iron acquisition, quorum sensing, phenazine biosynthesis, and extracellular proteolysis. At the same time, heterogeneity was observed in selected accessory or clinically relevant loci, most notably type III secretion system effector genes and selected pyoverdine-, pilus- and motility-associated genes. This pattern is consistent with the biology of *P. aeruginosa* as a highly adaptable opportunistic pathogen rather than a pathogen defined by a single virulence determinant [1,9,15,16].

The clinical relevance of these findings should be interpreted in the specific context of canine otitis externa. Otitis externa is a multifactorial disease in which primary, predisposing and perpetuating factors alter the ear-canal microenvironment and create conditions that favor secondary microbial overgrowth [1,2,3,4,10]. Previous reviews and clinical studies have emphasized that *P. aeruginosa* is particularly important in chronic, suppurative and treatment-refractory otitis externa, where it may persist in inflamed, moist and exudative niches and frequently forms biofilm [1,5,10]. Secker et al. similarly highlighted *Pseudomonas* spp. as major contributors to difficult canine otitis cases, especially because biofilm formation, intrinsic resistance and treatment-associated selection can interact in the ear canal [1]. Our genomic results support this clinical view: the near-universal presence of adhesion, motility, matrix-associated and secretion-system genes suggests that these isolates possess the genomic machinery required for colonization and persistence once the local ear environment becomes permissive.

The widespread detection of alginate-associated genes is particularly relevant. Alginate and related exopolysaccharide pathways are central to mucoid and biofilm-associated phenotypes, especially in chronic *P. aeruginosa* infections [9,17]. In our dataset, alginate structural and regulatory loci were broadly distributed, which is consistent with the concept that canine otitis-associated isolates retain the capacity to establish protected bacterial communities. This agrees with previous descriptions of *P. aeruginosa* biofilm biology, where biofilm growth reduces antimicrobial penetration, supports slow-growing and persister-like subpopulations, and increases tolerance to host defense mechanisms [6,9,17]. This interpretation is consistent with previous reviews emphasizing that biofilm-mediated adaptive resistance is a key survival strategy under antimicrobial exposure, because the extracellular matrix reduces diffusion, modifies the internal microenvironment and promotes persistence of metabolically less active cells [9,17]. Therefore, the conserved alginate and biofilm-associated repertoire observed in the present study should not be interpreted as a purely descriptive genomic finding, but as a plausible biological explanation for the chronicity and therapeutic difficulty of canine *P. aeruginosa*-associated otitis.

The broader phenotypic dataset from which the WGS subset was selected showed reduced susceptibility to several clinically relevant agents, including fluoroquinolones and aminoglycosides, thereby providing the resistance-related background for the present virulome-focused analysis. Previous studies from Croatia, Romania and the Midwestern United States provide useful regional and international context for interpreting these findings [19,33]. Mekić et al. examined canine otitis externa-derived *P. aeruginosa* isolates in Croatia and reported substantially more favorable ceftazidime and gentamicin susceptibility patterns than those observed in the Hungarian phenotypic dataset [19]. More recently, Popa et al. investigated antimicrobial susceptibility and fluoroquinolone resistance patterns of canine otitis externa-derived *P. aeruginosa* isolates in Romania, highlighting that treatment-relevant resistance phenotypes, particularly those affecting fluoroquinolone use, are also an important regional concern in Central and Eastern Europe [34]. KuKanich et al. reported higher susceptibility rates for amikacin, gentamicin, ceftazidime, piperacillin–tazobactam and imipenem in canine otitis-associated isolates from the Midwestern United States than those observed in the present Hungarian collection [33]. These phenotypic differences are not directly explained by the virulome, but they provide an important clinical background: the isolates analyzed here originate from a population in which resistance burden is already substantial. The present study therefore adds that these isolates are not only difficult because of antimicrobial susceptibility profiles, but also because they carry conserved genomic systems linked to persistence, tissue interaction and biofilm-associated survival.

The high prevalence of type IV pili and flagellar motility genes is consistent with previous work on *P. aeruginosa* pathogenesis. Type IV pili contribute to twitching motility, surface attachment and early biofilm development, while flagella support swimming motility, epithelial interaction and host immune stimulation [9,15,16]. In canine otitis externa, these systems are biologically plausible contributors to colonization of an inflamed epithelial surface covered by cerumen, exudate and microbial communities. Similar clinical reasoning has been used in discussions of otitis externa management, where physical cleaning of the ear canal is considered essential because debris and biofilm reduce the effectiveness of topical treatment [1,10]. The variability of selected pilus and flagellar loci in our dataset may reflect lineage-level diversity, annotation differences or accessory genomic variation. However, it also identifies candidate genes for future studies linking virulome patterns with clinical severity, recurrence, otitis media involvement or treatment outcome.

The type III secretion system effector results represent one of the most important comparative findings of this study. The T3SS core machinery was broadly conserved, but effector carriage was heterogeneous: *exoT* and *exoY* were frequent, *exoS* was detected in most isolates, and *exoU* was present in a smaller but clinically relevant subset. This is consistent with the broader *P. aeruginosa* literature, where *exoS* and *exoU* are commonly interpreted as markers of different pathogenic strategies. *exoS*-positive strains are often associated with invasive and persistent phenotypes, whereas *exoU*-positive strains are frequently linked to acute cytotoxicity and more severe tissue damage in human-pathogen studies [16]. In the present canine otitis collection, the predominance of *exoS* over *exoU* is compatible with a higher frequency of genomic backgrounds commonly associated with invasive or persistent phenotypes than of backgrounds carrying the cytotoxicity-associated *exoU* determinant. Nevertheless, the detection of *exoU* in 15/70 isolates identifies a subset that may warrant further functional investigation, but it should not be interpreted as evidence of demonstrated cytotoxicity. This interpretation is hypothesis-generating rather than confirmatory, because the present study assessed gene presence only and did not evaluate T3SS expression, effector secretion or host-cell injury. Functional cytotoxicity assays, epithelial-cell models or transcriptomic analysis under ear-canal-like conditions would be required to determine whether these *exoU*-positive isolates display increased tissue-damaging behavior.

The broad distribution of siderophore-associated genes also fits well with the current understanding of *P. aeruginosa* infection biology. Iron is restricted in host tissues, and *P. aeruginosa* relies on high-affinity siderophore systems such as pyoverdine and pyochelin to acquire iron during infection [9,15]. In our dataset, pyochelin-associated genes were highly conserved, while selected pyoverdine-related loci showed greater variability. This differs from a simple “all-or-nothing” view of siderophore carriage and suggests that canine otitis-associated isolates may share a core iron-acquisition background while differing in accessory or receptor-level siderophore components. This is relevant because iron acquisition is linked not only to growth in host-restricted environments, but also to oxidative stress tolerance, interbacterial competition, biofilm formation and virulence regulation. The presence of these systems therefore supports the interpretation that canine otitis isolates are equipped to persist in inflammatory niches where free iron availability is limited.

The population-structure analyses indicated a genetically diverse isolate collection rather than a single clonal expansion. MLST identified multiple known sequence types but also a high number of novel or incomplete profiles, which required cautious interpretation. For this reason, population-structure inference was strengthened by core-genome phylogenetic analysis. The core-genome tree confirmed that the isolates were distributed across multiple phylogenetic branches rather than forming a single outbreak-like cluster. This supports the interpretation that the conserved virulence-associated genomic backbone observed in the study is not merely the result of one locally expanded lineage but is present across diverse canine otitis-associated *P. aeruginosa* backgrounds. The distribution of *exoU*-positive isolates across the phylogeny further suggests that this effector profile was not restricted to a single dominant clonal background. Nevertheless, the high proportion of novel or incomplete MLST profiles remains a limitation of sequence-type-based interpretation and may reflect allelic novelty, database underrepresentation or locus-level assembly uncertainty [7,18]. Elfadadny et al. reported genetically diverse *P. aeruginosa* isolates from canine ear canals in Japan and emphasized the value of genotyping for understanding potential transmission and risk patterns in companion animals [18]. Haenni et al. similarly described a diverse population structure among animal infection-derived *P. aeruginosa* isolates in France [7]. Our results are compatible with these observations: although the virulence backbone was highly conserved, the MLST profile was heterogeneous and included multiple sequence types as well as many novel or incomplete calls. This combination is important. It suggests that the conserved virulence architecture is not merely the result of one locally expanded lineage but may be broadly distributed across diverse canine otitis-associated *P. aeruginosa* backgrounds. At the same time, the high proportion of novel or incomplete MLST calls should be interpreted cautiously, because these profiles may reflect genuine allelic novelty, assembly fragmentation, missing loci or database limitations.

The weak relationship between virulome size and modified MAR index is another important result. Total VFDB gene count was not strongly correlated with phenotypic resistance burden, and *exoU*-positive isolates did not show a higher modified MAR index than *exoU*-negative isolates. This agrees with the broader conceptual distinction between antimicrobial resistance and virulence: both contribute to clinical risk, but they are not interchangeable biological dimensions. A highly resistant isolate is not necessarily the most virulence-gene-rich isolate, and an isolate carrying a potentially cytotoxic effector gene is not necessarily the most resistant by MIC criteria. This distinction is especially relevant for companion-animal infections, where treatment failure may reflect the combined effects of resistance, biofilm tolerance, local inflammation, canal anatomy, exudate, owner compliance and primary dermatological disease. Recent Hungarian and regional veterinary AMR studies further support the value of integrated surveillance frameworks that combine phenotypic and genomic information rather than relying on single isolate-level resistance classifications alone [35,36,37,38,39].

The present results should also be interpreted alongside the resistance-related genomic and phenotypic background of canine otitis-associated *P. aeruginosa*. Although this study focused on virulence-associated determinants rather than antimicrobial resistance genes, the same clinical problem is shaped by both dimensions. In *P. aeruginosa*, intrinsic and acquired resistance mechanisms, including low outer-membrane permeability, efflux systems, β-lactamases, aminoglycoside-modifying enzymes, lipid A modification and fluoroquinolone-associated mechanisms, may restrict therapeutic options and contribute to treatment failure [15,30]. At the same time, the present virulome analysis shows that canine otitis-associated isolates also carry conserved systems related to adhesion, motility, biofilm formation, secretion, iron acquisition and extracellular tissue damage. Therefore, resistance and virulence should not be evaluated as separate or competing explanations of clinical difficulty, but as complementary components of pathogenic potential. This strengthens the argument that future veterinary surveillance should jointly evaluate phenotypic susceptibility, resistance determinants, virulence systems and, where relevant, mobile genetic elements. Such integration is especially important in a One Health context, because companion animals live in close contact with humans and may share household, clinical and environmental microbial reservoirs [18,36,40].

From a therapeutic perspective, the present virulome findings provide a biological rationale for why susceptibility testing alone may not fully predict treatment outcome in canine otitis externa. Topical preparations can reach concentrations that exceed planktonic MIC values, but biofilm matrix, exudate, cerumen, local pH, tissue inflammation and poor penetration may still reduce efficacy [6,10,17]. This is particularly relevant for *P. aeruginosa*, where biofilm formation and adaptive resistance can reduce antimicrobial susceptibility without requiring stable acquisition of new resistance genes [9,17]. The detection of alginate, quorum-sensing, motility, siderophore and secreted enzyme systems in the present isolates supports this interpretation. Therefore, in clinical practice, antimicrobial selection should be accompanied by ear cleaning, management of the primary dermatological disease, evaluation of otitis media, and consideration of adjunctive anti-biofilm strategies where appropriate [1,10,20].

These findings may also inform future hypothesis-driven studies of adjunctive treatment and topical product-development strategies. However, the present genomic dataset does not directly test treatment efficacy, biofilm disruption, antimicrobial penetration or clinical outcome; therefore, these translational implications should be interpreted cautiously. For canine *P. aeruginosa* otitis, approaches targeting planktonic susceptibility alone may be insufficient if biofilm formation, outer-membrane protection, quorum-sensing-regulated behavior and limited antimicrobial penetration contribute to persistence [41,42,43]. Several adjunctive strategies are relevant for future testing in this context. Tris-EDTA can damage the bacterial outer membrane and enhance the activity of selected antimicrobials, particularly aminoglycosides and fluoroquinolones [1,42]. Geraniol and other essential-oil components have been investigated for antibacterial, efflux-modulating or anti-biofilm activity, although ototoxicity and achievable local concentrations must be considered carefully [43,44,45]. Sebők et al. also reviewed antimicrobial peptides as emerging anti-infective tools with direct antimicrobial and immunomodulatory properties [44]. The main translational value of the present virulome data is that they may help select genetically representative challenge isolates for future functional or formulation studies: rather than relying on a single reference strain, downstream experiments should include isolates with different *exoS*/*exoU* profiles, siderophore patterns, virulence-gene counts, phylogenetic backgrounds and resistance-burden levels.

Several limitations should be acknowledged. First, the WGS subset was deliberately selected from the phenotypic collection to capture antimicrobial susceptibility and resistance-burden diversity and should not be interpreted as a random or prevalence-representative sample of all canine otitis-associated *P. aeruginosa* isolates in Hungary. This selection strategy may have influenced the observed prevalence of selected virulence-associated determinants, particularly if resistance-burden profiles and lineage backgrounds are not evenly distributed in the broader isolate population. Therefore, prevalence estimates should be interpreted as descriptive for the sequenced subset rather than as national prevalence estimates. Second, virulence-gene detection does not prove gene expression, secretion, biofilm biomass, cytotoxicity or clinical severity. In addition, selected loci were not independently confirmed by PCR in this study. Although the long-read WGS approach provides genome-wide sequence-based evidence, targeted PCR, RT-qPCR, transcriptomics, proteomics, biofilm assays, cytotoxicity assays or ear-canal-mimicking in vitro systems would be useful to validate selected loci and determine their functional relevance under otitis-relevant conditions. Third, detailed clinical metadata, including chronicity, previous antimicrobial exposure, otoscopic findings, otitis media status, cleaning protocol, treatment choice and clinical outcome, were not available for integrated analysis. Finally, the high proportion of novel or incomplete MLST calls remains a limitation of sequence-type-based interpretation, because these profiles may reflect genuine allelic novelty, assembly fragmentation, missing loci or database limitations.

Despite these limitations, this study adds a clinically meaningful layer to the companion MIC-focused analysis. Previous canine otitis studies have mainly emphasized antimicrobial susceptibility, resistance patterns or population structure [7,18,19,33]. The present analysis shows that canine otitis-associated *P. aeruginosa* isolates should also be evaluated through their virulence-associated genomic architecture. The isolates carried a conserved set of systems that plausibly contribute to adhesion, motility, biofilm formation, iron acquisition, extracellular tissue damage and persistence, while accessory variation in T3SS effectors and selected surface or siderophore genes may influence isolate-level pathogenic behavior. Future veterinary surveillance should therefore integrate MIC profiles, resistance genes, virulence architecture, mobile genetic elements and clinical metadata. Such an integrated approach would better identify isolate profiles associated with recurrent, severe or treatment-refractory disease and may support rational development of topical and adjunctive therapeutic strategies for canine *P. aeruginosa*-associated otitis.

## 5. Conclusions

Canine otitis externa-derived *Pseudomonas aeruginosa* isolates from this Hungarian collection carried a conserved and diverse predicted virulence-associated genomic repertoire, including systems involved in adhesion, motility, biofilm matrix formation, secretion, iron acquisition, extracellular enzyme activity and T3SS-mediated effector delivery. Core-genome phylogenetic analysis confirmed that the sequenced isolates represented a genetically heterogeneous clinical collection rather than a single clonal expansion. The presence of an *exoU*-positive subset and the weak relationship between virulome size and resistance burden suggest that predicted virulence-associated genomic potential and antimicrobial resistance should be evaluated as complementary dimensions. However, gene detection alone does not demonstrate expression, functional virulence or clinical severity. This dataset supports integrated genomic surveillance frameworks for companion-animal infections, while future targeted and functional validation will be needed to confirm the biological activity and clinical relevance of selected loci.

## Figures and Tables

**Table 1:** Dataset and assembly-quality summary.

Metric	Value
Phenotypic isolate collection	110 isolates
Selected WGS subset	70 isolates
Virulome-analyzed WGS subset	70 isolates
Median genome size	6.57 Mbp
Median GC content	66.27%
Median CheckM completeness	99.68%
Median CheckM contamination	0.14%

**Table 2:** Virulence-associated functional systems detected in the whole-genome sequenced subset.

Virulence-Associated Functional System	Distinct Genes	Isolates	Prevalence (%)
Type IV pili/twitching motility	33	70	100.00
Flagellar motility	30	70	100.00
Type VI secretion system	26	70	100.00
Alginate and mucoid biofilm matrix	22	70	100.00
Other/accessory virulence-associated loci	17	70	100.00
Flagellar/glycan-associated motility antigens	16	70	100.00
Pyochelin siderophore system	11	70	100.00
Xcp type II secretion system	11	70	100.00
Phenazine biosynthesis	8	70	100.00
LPS and surface antigen structure	7	70	100.00
Pyoverdine siderophore system	5	70	100.00
Alginate regulation	4	70	100.00
Rhamnolipid biosurfactant system	3	70	100.00
Secreted proteases and tissue-damaging enzymes	3	70	100.00
Phenazine/pyocyanin system	2	70	100.00
Quorum sensing	2	70	100.00
Phospholipase C	1	70	100.00
Type III secretion system	36	68	97.14
T3SS effector ExoT	1	68	97.14
Exotoxin A	1	66	94.29

**Table 3:** Clinically relevant or variable virulence-associated genes.

Gene	Functional System	Isolates	Prevalence (%)
*algD*	Alginate and mucoid biofilm matrix	70	100.00
*algU*	Alginate and mucoid biofilm matrix	70	100.00
*aprA*	Secreted proteases and tissue-damaging enzymes	70	100.00
*clpV1*	Type VI secretion system	70	100.00
*pchA*	Pyochelin siderophore system	70	100.00
*mucB*	Alginate and mucoid biofilm matrix	70	100.00
*mucA*	Alginate and mucoid biofilm matrix	70	100.00
*lasB*	Secreted proteases and tissue-damaging enzymes	70	100.00
*lasA*	Secreted proteases and tissue-damaging enzymes	70	100.00
*icmF1/tssM1*	Type VI secretion system	70	100.00
*hcp1*	Type VI secretion system	70	100.00
*rhlA*	Rhamnolipid biosurfactant system	70	100.00
*rhlB*	Rhamnolipid biosurfactant system	70	100.00
*plcH*	Phospholipase C	70	100.00
*phzS*	Phenazine/pyocyanin system	70	100.00
*phzM*	Phenazine/pyocyanin system	70	100.00
*pchD*	Pyochelin siderophore system	70	100.00
*pchC*	Pyochelin siderophore system	70	100.00
*pchB*	Pyochelin siderophore system	70	100.00
*rhlI*	Quorum sensing	70	100.00
*rhlC*	Rhamnolipid biosurfactant system	68	97.14
*exoT*	T3SS effector ExoT	68	97.14
*pvdA*	Pyoverdine siderophore system	67	95.71
*toxA*	Exotoxin A	66	94.29
*lasI*	Quorum sensing	64	91.43
*exoY*	T3SS effector ExoY	63	90.00
*pilY1*	Type IV pili/twitching motility	60	85.71
*exoS*	T3SS effector ExoS	50	71.43
*fliC*	Flagellar motility	33	47.14
*fliD*	Flagellar motility	33	47.14
*fpvA*	Pyoverdine siderophore system	29	41.43
*pvdE*	Pyoverdine siderophore system	29	41.43
*pvdD*	Pyoverdine siderophore system	25	35.71
*exoU*	T3SS effector ExoU	15	21.43
*pilA*	Type IV pili/twitching motility	8	11.43

**Table 4:** MLST-based population structure in the whole-genome sequencing (WGS) subset.

MLST Sequence Type	Number of Isolates
novel/incomplete	43
253	3
1076	2
94	2
611	2
200	2
654	2
1182	1
1203	1
2048	1
111	1
2498	1
2333	1
2222	1
217	1
207	1
275	1
298	1
3300	1
557	1

**Table 5:** Exploratory integration of virulence features with phenotypic resistance burden.

Comparison	Method	Estimate	*p* Value/Proportion
Total VFDB gene count vs. modified MAR index	Spearman correlation	ρ = −0.05	*p* = 0.687
*exoU*-positive vs. *exoU*-negative modified MAR index	Mann–Whitney U test	median 0.56 vs. 0.56	*p* = 0.549
*exoU* prevalence	Descriptive	15/70	21.43%
*exoS* prevalence	Descriptive	50/70	71.43%

## Data Availability

The whole-genome sequencing data generated in this study have been deposited in the NCBI database under BioProject accession number PRJNA1475700. The phenotypic dataset supporting the findings of this study is available from the corresponding author upon reasonable request.
